# Unlocking electronic health records: a hybrid graph RAG approach to safe clinical AI for patient QA

**DOI:** 10.3389/fdgth.2026.1780700

**Published:** 2026-03-11

**Authors:** Samuel Thio, Matthew Lewis, Spiros Denaxas, Richard J. B. Dobson

**Affiliations:** 1 Institute of Health Informatics, University College London, London, United Kingdom; 2 Department of Biostatistics and Health Informatics, King’s College London, London, United Kingdom; 3 DRIVE-Health CDT, UKRI Engineering and Physical Sciences Research Council, London, United Kingdom; 4 Computational Medicine Research Lab, Interdisciplinary Transformation University (IT:U), Linz, Austria; 5 Interdisciplinary Transformation University (IT:U), Linz, Austria; 6 British Heart Foundation Data Science Centre, London, United Kingdom; 7 National and Kapodistrian University of Athens, Athens, Greece; 8 NIHR Biomedical Research Centre, South London and Maudsley NHS Foundation Trust and King's College London, London, United Kingdom; 9 NIHR Biomedical Research Centre, University College London Hospitals NHS Foundation Trust, London, United Kingdom; 10 Health Data Research UK London, University College London, London, United Kingdom

**Keywords:** electronic health records, knowledge graphs, large language models, natural language processing, Neo4j, retrieval-augmented generation, Text2Cypher, vector embeddings

## Abstract

**Introduction:**

Electronic health record (EHR) systems present clinicians with vast repositories of clinical information, creating a significant cognitive burden where critical details are easily overlooked. While Large Language Models (LLMs) offer transformative potential for data processing, they face significant limitations in clinical settings, particularly regarding context grounding and hallucinations. Current solutions typically isolate retrieval methods, focusing either on structured data (SQL/Cypher) or unstructured semantic search, but fail to integrate both simultaneously.

**Methods:**

This work presents MediGRAF (Medical Graph Retrieval Augmented Framework), a novel hybrid Graph RAG system that bridges this gap. By uniquely combining Neo4j Text2Cypher capabilities for structured relationship traversal with vector embeddings for unstructured narrative retrieval, MediGRAF enables natural language querying of the complete patient journey. The system was evaluated using data from 10 patients from the MIMIC-IV dataset, generating 5,973 nodes and 5,963 relationships, across varying query complexities using both deterministic retrieval metrics and a structured clinical expert evaluation protocol.

**Results:**

The system demonstrated 100% recall for factual queries, meaning all relevant information was retrieved and included in the output. Complex inference tasks achieved a mean expert quality score of 4.25/5 with zero safety violations across all evaluated cases.

**Discussion:**

These results demonstrate that hybrid graph-grounding significantly advances clinical information retrieval, offering a safer and more comprehensive alternative to standard LLM deployments. By combining structured graph traversal with semantic vector search, MediGRAF addresses the critical limitations of isolated retrieval approaches, establishing a foundation for responsible AI deployment in clinical settings.

## 1 Introduction

The proliferation of electronic health record (EHR) systems has fundamentally transformed healthcare delivery, creating comprehensive digital repositories of patient information encompassing both structured data elements (medications, laboratory results, vital signs) and unstructured free-text narratives (clinical notes, discharge summaries). However, this wealth of information presents a paradoxical challenge: while more data are available than ever before, manually reviewing current and historical admission notes has become increasingly time-consuming and cognitively demanding, potentially leading to overlooked clinically significant information (1). This information overload contributes directly to clinician burnout and may compromise patient safety when critical details are missed during time-pressured clinical encounters.

Traditional approaches to managing EHR data have proven inadequate in capturing the complex temporal and relational aspects inherent in healthcare information. Conventional relational databases, while efficient for storing structured data, struggle to represent the intricate relationships between clinical events, medications, diagnoses, and patient outcomes (2). These relationships embody not only associations but also causal chains, temporal dependencies, and clinical reasoning pathways fundamental to understanding a patient’s health trajectory (3, 4).

Recent advances in large language models (LLMs) have demonstrated transformative potential for natural language processing in healthcare (5, 6). However, when applied to patient-specific data retrieval, LLMs face significant limitations, most notably lacking necessary context grounding, leading to hallucinations (plausible sounding but factually incorrect information) (7, 8).

Graph databases have emerged as a promising solution. Unlike traditional relational databases with rigid tables and predefined relationships, graph databases represent information as networks of nodes and edges, naturally capturing the complex web of relationships characterizing healthcare data. Neo4j has demonstrated particular advantages over traditional SQL databases in healthcare applications, offering superior performance for complex traversal queries (9).

Retrieval-Augmented Generation (RAG) has gained attention as a method to enhance LLM capabilities by grounding responses in retrieved contextual information (10). Graph RAG represents an evolution specifically designed to leverage graph database structural advantages. Recent work on hybrid RAG architectures has demonstrated effectiveness of combining multiple retrieval modalities (11), with approaches integrating knowledge graphs with vector retrieval showing superior performance.

Despite these advances, a significant gap remains: no existing system successfully integrates graph database technology with both Text2Cypher capabilities and vector embeddings to create a unified solution for querying patient-level EHR data. Current approaches typically focus on either structured data retrieval or semantic search, but not both simultaneously. This study addresses this gap by developing and evaluating MediGRAF (Medical Graph Retrieval Augmented Framework), a novel Graph RAG system that combines Neo4j graph database technology with large language models to enable natural language querying of complex clinical data. We present an optimized graph schema for EHR representation, implement Text2Cypher translation for accessible graph querying, and integrate vector embeddings for semantic search across unstructured clinical narratives. Our evaluation using MIMIC-IV data demonstrates MediGRAF’s ability to achieve perfect recall for factual queries while maintaining high performance and safety standards for complex clinical inference tasks, establishing a foundation for next-generation clinical information retrieval systems.

### 1.1 Significance and clinical integration

The significance of this research extends beyond technical innovation to address pressing clinical needs. By enabling natural language querying of complex EHR data, this system has the potential to reduce the cognitive burden on clinicians, improve the efficiency of clinical information retrieval, and ultimately enhance patient care quality. The approach is particularly relevant for the NHS context, where time pressures and resource constraints make efficient information access critical for maintaining care quality while managing increasing patient volumes.

Furthermore, the system is architected to align with existing NHS informatics infrastructure, offering a clear pathway for integration with NLP platforms like CogStack. As detailed in Section 5.1, this integration allows MediGRAF to leverage backend ingestion pipelines for live, real-time clinical decision support.

## 2 Related work

The development of the MediGRAF system sits at the intersection of three rapidly evolving domains: graph database management in healthcare, natural language processing (NLP) via Large Language Models (LLMs), and hybrid information retrieval strategies. This section reviews the progression of these technologies, identifying the specific technological gaps that this research aims to address.

### 2.1 Graph databases in healthcare data management

Traditional approaches to managing Electronic Health Records (EHR) have largely relied on relational database management systems (RDBMS) (12). While efficient for transactional data, these systems struggle to represent the complex, interconnected nature of clinical events (2). The rigid tabular structure of RDBMS fails to naturally capture the causal chains, temporal dependencies, and clinical reasoning pathways that define a patient’s health trajectory (13).

Graph databases have emerged as a superior alternative by representing data as networks of nodes and edges. This structure aligns more closely with biological and clinical reality (14). In a foundational study establishing the feasibility of this approach, Stothers and Nguyen demonstrated that Neo4j offered distinct advantages over PostgreSQL for healthcare applications (9). Their work highlighted a specific use case: handling complex traversal queries such as linking patients to diagnoses and treatments across multiple encounters where graph databases significantly outperformed relational counterparts in both speed and query intuitiveness. This evidence suggests that graph databases are better suited to handle the scale and relational complexity of real-world EHR data.

### 2.2 Large language models and the context gap

Parallel to advances in database technology, the rise of Transformers and Large Language Models (LLMs) has transformed clinical NLP (5). Models such as GPT-4 have demonstrated the ability to process unstructured narratives that make up a significant portion of EHR data (6, 15). However, the deployment of LLMs in patient-specific retrieval tasks faces a critical hurdle: the lack of contextual grounding.

Research by Zubiaga and others highlights that without access to external veridical knowledge, LLMs are prone to “hallucinations” generating plausible but factually incorrect information (7, 8, 16, 17). In a clinical setting, where precision is paramount, this limitation is prohibitive. While LLMs excel at linguistic fluency, they lack an inherent “memory” of the specific patient’s history, necessitating external retrieval mechanisms to ground their outputs in factual records.

### 2.3 Retrieval-augmented generation (RAG) and GraphRAG

To mitigate hallucination risks, Lewis et al. introduced Retrieval-Augmented Generation (RAG), a framework that retrieves relevant documents to condition the LLM’s generation (10). In the medical domain, RAG has demonstrated particular utility for evidence-based answering (18, 19). However, standard RAG often misses the structural relationships inherent in medical data (9).

This limitation led to the development of “Graph RAG,” which leverages the structural advantages of knowledge graphs. Edge et al. applied this to the use case of query-focused summarisation, demonstrating that graph structures could improve the relevance and coherence of generated summaries by capturing global relationships that vector-only retrieval might miss (20). Similarly, Wu et al. demonstrated that Medical Graph RAG could improve safety in medical LLMs by incorporating structured clinical relationships alongside narrative data (21).

### 2.4 Bridging the interaction gap: Text2Cypher

A significant barrier to the adoption of graph databases in clinical practice is the technical expertise required to query them. The Cypher query language, while powerful, is inaccessible to most clinicians. To address this, current research leverages the code-generation capabilities of modern LLMs (22). Building on foundational advancements in Text-to-SQL and semantic parsing (23, 24), recent methodologies have adapted these techniques to the graph domain. This “Text2Cypher” approach utilises LLMs to translate natural language questions directly into executable Cypher queries (25). This innovation is crucial for the clinical utility of the proposed system, as it allows end-users to interact with complex graph structures using standard medical terminology without needing to learn query syntax.

### 2.5 The case for hybrid retrieval architectures

While Graph RAG and Text2Cypher tackle structured data and accessibility respectively, they often struggle with the free-text narratives (discharge summaries, radiology reports) that contain vital clinical nuance (26). Recent work on “Hybrid RAG” architectures has attempted to bridge this divide (19, 20, 26). Sarmah et al. demonstrated that integrating knowledge graphs with vector retrieval (semantic search) leads to superior performance in information extraction tasks compared to using either modality in isolation (11).

In this context, and consistent with recent Information Retrieval literature (27, 28), we explicitly define “semantic search” as dense vector retrieval, where queries and documents are mapped to a continuous embedding space to identify conceptual similarity in unstructured text. This modality is distinct from, and complementary to, the explicit structural traversal performed by Cypher on the knowledge graph.

However, a gap remains in applying these hybrid architectures to patient-level EHR data. Current approaches typically focus on either structured data retrieval (via Text2Cypher/Text2SQL) (25, 29) or semantic search (via vectors) (18), but rarely integrate both into a unified pipeline. This study addresses this gap by proposing a system that combines the precision of Text2Cypher for structured facts with the semantic reach of vector embeddings for clinical narratives, aiming to provide a comprehensive view of the patient journey.

## 3 Methodology

### 3.1 Data source and preprocessing

We utilised the MIMIC-IV dataset (version 3.1) (30, 31), a freely accessible, de-identified electronic health record database from Beth Israel Deaconess Medical Center. Access required CITI training completion and PhysioNet data use agreement. All guidelines for responsible AI use with MIMIC data were followed and no re-identification attempts were made.

Using data from ten patients, we generated 5,973 nodes and 5,963 relationships, creating a dataset sufficient for evaluating patient-level question answering (QA) across varying levels of difficulty. These 10 patients were selected based on specific inclusion criteria designed to evaluate the system across a spectrum of clinical and temporal complexity:

- **Variable Temporal Complexity:** The cohort was stratified to include both single-encounter patients (n=4) to evaluate baseline cross-sectional retrieval, and multi-encounter patients (n=6, range 2–9 admissions) to stress-test the graph’s ability to perform longitudinal reasoning and temporal tracking.
- **Unstructured Data Availability:** Inclusion required the presence of both Radiology & Discharge notes to test the hybrid retrieval of unstructured narratives alongside structured codes.
- **Diverse Specialities:** Patients were selected to represent a broad range of medical domains (Table 1) to ensure the embedding model generalises across different medical vocabularies (e.g., cardiology vs. oncology terminology).

**Table 1:** Cohort characteristics: selected MIMIC-IV patients (n=10).

Subject ID	Admissions	Notes${}^{a}$	Primary clinical focus (simplified)
Single-Encounter (Baseline Complexity)			
10461137	1	4	Pulmonary Fibrosis/Diastolic Heart Failure
12017557	1	2	Infectious Colitis/Gastroenteritis
14419899	1	7	Orthopedic (Femur Fracture)
14968093	1	2	HIV Disease Management
Multi-Encounter (Longitudinal Complexity)			
11578849	4	8	Asthma (Acute Exacerbation)/COPD
11857644	2	10	Oncology (Brain/Spine Neoplasm)
12522208	9	30	Complex: Diabetes (Type 2), CKD Stage V, Epilepsy
14037695	4	8	Cardiology (Valvular Disease)/Acute Kidney Failure
17595300	3	11	Respiratory (Pneumonitis)/Spinal Disc Displacement
19801500	2	7	Sepsis/Aortic Stenosis

The cohort includes both single-encounter patients and multi-encounter patients.

${}^{a}$Total count of Discharge Summaries and Radiology Reports combined.

**Figure 1:**
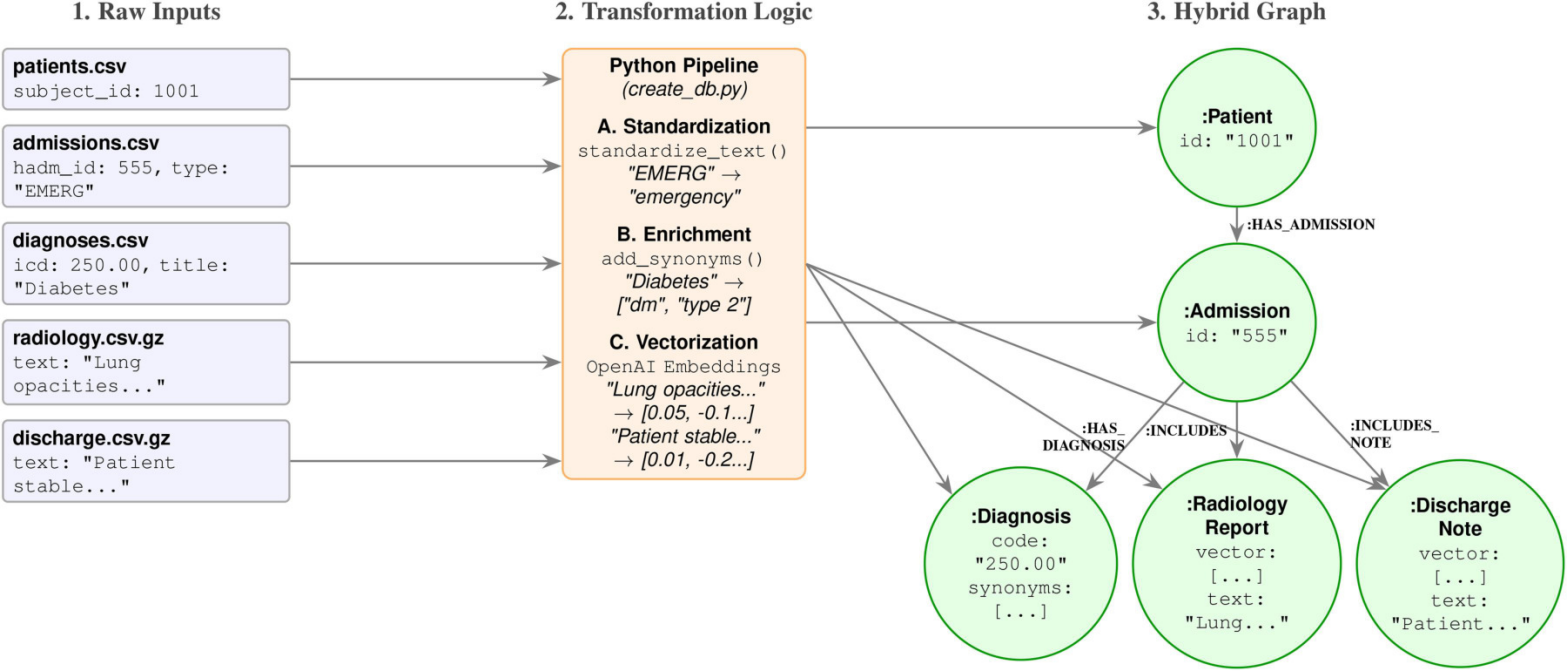
Comprehensive transformation pipeline. The diagram illustrates how heterogeneous data sources are processed: structured data (Diagnoses) undergoes synonym enrichment, while unstructured narratives (Radiology Reports, Discharge Notes) undergo vector embedding. All entities are linked via the central Admission node, enabling hybrid retrieval. Note: Other structured entities (e.g., Lab Events, Medications) undergo similar standardization but are omitted here for visual clarity.

Data preprocessing followed a systematic pipeline to transform raw tabular records into a connected graph structure. As illustrated in Figure 1, data elements were extracted from disparate MIMIC-IV tables, including structured clinical events (diagnoses, procedures, lab events, medications) and unstructured narratives (radiology reports, discharge summaries). We applied a standardization pipeline to all categorical text fields: a standardize_text function converted inputs (e.g., medication routes, admission types) to lowercase, and a semantic enrichment function injected synonyms into diagnosis nodes (e.g., adding “dm” to “diabetes”). For unstructured data, discharge notes and radiology reports were processed to generate vector embeddings as node properties. Crucially, the relational keys in the raw CSVs (e.g., subject_id, hadm_id) were utilized to link disparate entities into a unified, multi-modal subgraph for each patient.

### 3.2 Graph database schema design

The development of an optimised graph schema for Neo4j required careful consideration of both clinical relationships and query performance. The schema represents eight primary node types, capturing distinct clinical entities as detailed in Table 2.

**Table 2:** Schema specification: node labels and key attributes.

Node label	Description	Key attributes (properties)
:Patient	Central anchor for patient history	subject_id*, gender, anchor_age, anchor_year
:Admission	distinct hospital encounter	hadm_id*, admission_type, admittime, dischtime
:Diagnosis	ICD-10 coded condition	icd_code*, long_title, synonyms (List)
:Procedure	Clinical intervention	icd_code*, long_title, chartdate
:LabEvent	Laboratory test result	itemid*, label, value, flag (e.g., “abnormal”)
:Medication	Prescribed pharmaceutical	medication*, route, frequency, verifiedtime
:DischargeNote	Full clinical summary	note_id*, text, embedding (Vector), charttime
:RadiologyReport	Imaging study report	note_id*, text, embedding (Vector), charttime

Properties marked with * are indexed for faster retrieval.

#### 3.2.1 Topology and isolation

**Figure 2:**
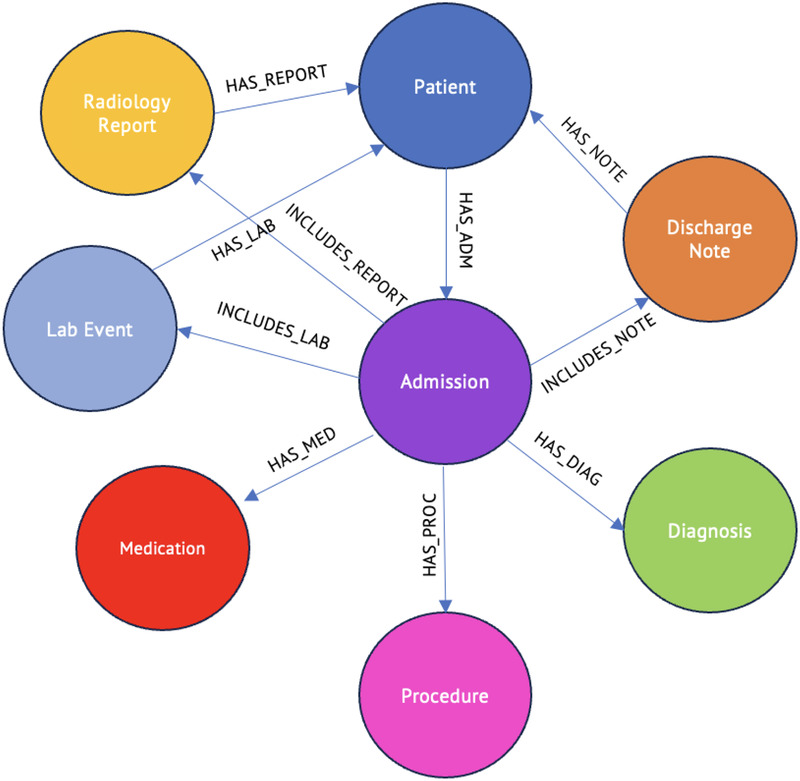
Neo4j schema of MIMIC-IV data with clinical nodes and relationships.

Crucially, the graph is structured as a collection of disconnected patient subgraphs (Figure 2). Clinical entities are not shared across patients; for example, if two patients are diagnosed with “Pneumonia,” two distinct :Diagnosis nodes are created, each linked to its respective :Admission. This design isolates patient contexts, preventing data leakage between subjects and simplifying compliance with data privacy deletion requests while focusing on patient level instead of population level queries.

Relationships between nodes were carefully defined to preserve clinical semantics and enable meaningful traversal queries. The HAS_ADMISSION relationship connects patients to their hospital admissions, maintaining temporal ordering. HAS_DIAGNOSIS links admissions to diagnosed conditions, preserving the context of when diagnoses were made. HAS_PROCEDURE connects admissions to performed procedures, while HAS_MEDICATION tracks prescribed medications. INCLUDES_LAB relationships link admissions to laboratory results, explicitly capturing the temporal sequence of tests during a hospital stay.

### 3.3 Vector embedding implementation

Free-text clinical documents, including discharge summaries and radiology reports, were processed using OpenAI’s text-embedding-3-small model, which generates 1,536-dimensional vector representations optimised for semantic similarity search (32). The embedding process involved several stages to ensure optimal representation of clinical content. Documents were first segmented into semantically coherent chunks, with chunk boundaries determined by clinical section headers and paragraph structures. Each chunk was embedded independently, with metadata preserved to maintain document provenance. The resulting embeddings were stored as properties of the relevant document nodes in the Neo4j database, enabling hybrid querying that combines graph traversal with vector similarity search using cosine similarity scores.

Let A denote the embedded query and B denote the embedded retrieved answer. The cosine similarity is then defined as (Equation 1):

$$\text{cosine\_similarity}\;(A,B)=\frac{A\cdot B}{\left\|A\|\|B\right\|}$$ (1)

Vector indexes were created using Hierarchical Navigable Small World (HNSW) indexing (33), which provides efficient approximate nearest neighbour search. Cosine similarity was selected as the similarity measure based on its effectiveness for text embeddings and widespread adoption in clinical NLP applications (34, 35).

### 3.4 MediGRAF pipeline architecture

**Figure 3:**
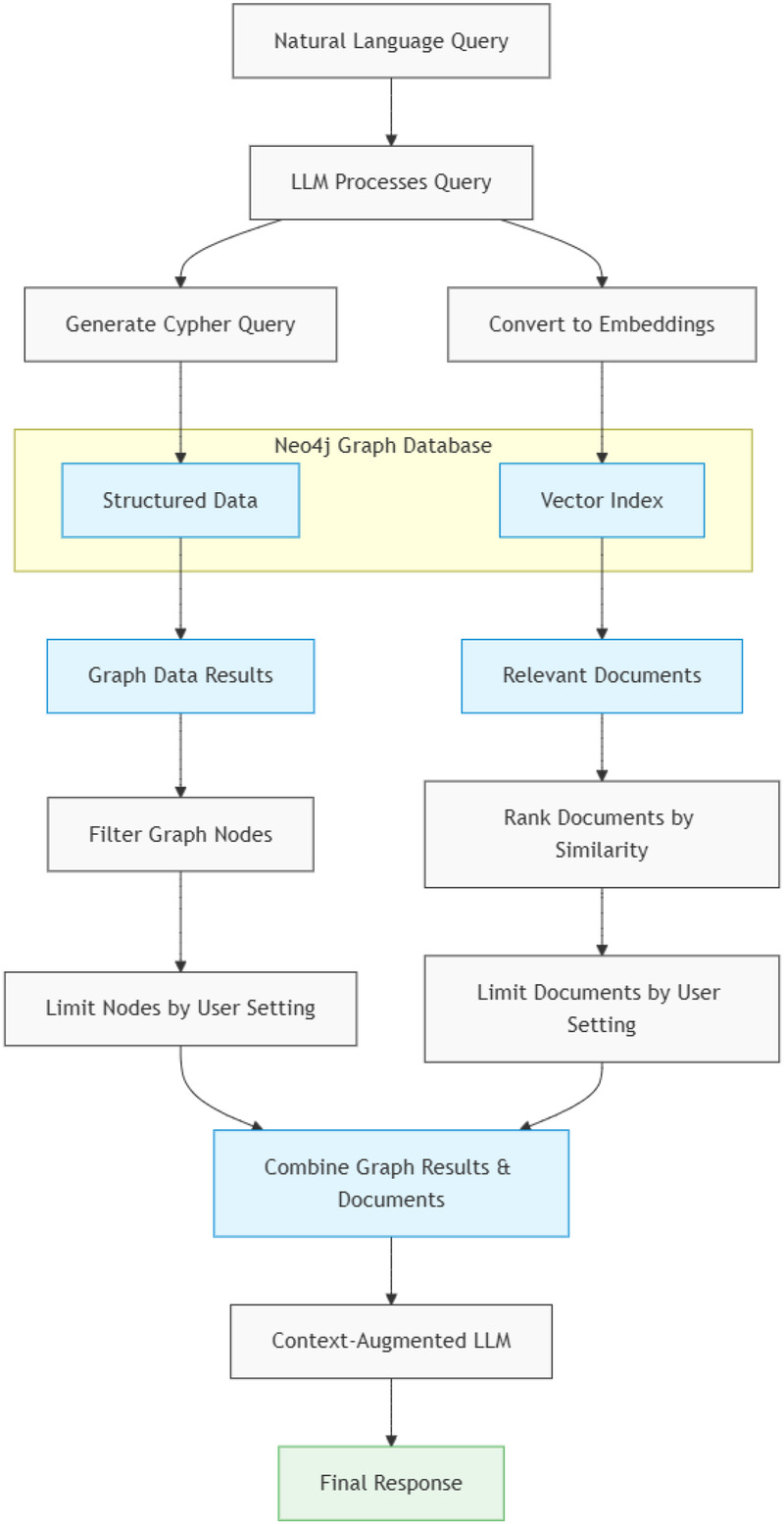
A workflow diagram showing the MediGRAF architecture from query input through graph retrieval to LLM augmentation and response generation.

The MediGRAF system integrates multiple components to process natural language queries and generate contextually grounded responses (Figure 3). The pipeline begins with query processing, where natural language input from clinicians undergoes initial analysis to identify key entities, temporal constraints, and query intent.

The Text2Cypher component leverages GPT-4o-mini with carefully engineered prompts to translate natural language queries into Cypher query language (25, 36). To ensure adherence to PhysioNet’s guidelines regarding the use of MIMIC-IV data with Large Language Models, inference was conducted using the Azure OpenAI Service Enterprise edition. In accordance with the guidelines for responsible use of MIMIC data (37), this infrastructure enforces a zero-day data retention policy and guarantees that input data is neither stored by the model provider nor utilized for model training. The prompt engineering process involved iterative refinement based on common clinical query patterns. As an illustration, the natural language query.

What did the radiology reports show for patient 10461137?

is translated into the following Cypher query:

MATCH (p:Patient {subject_id: '10461137'})-[:HAS_ADMISSION]->(a:Admission)-[:INCLUDES_RADIOLOGY_REPORT]->(r:RadiologyReport)
  RETURN r.note_id, r.text

Following the initial translation, the hybrid engine orchestrates the full retrieval and generation process via a four-step pipeline:

1. **Structured Retrieval:** The generated Cypher query is executed against the Neo4j database to extract structured nodes and relationships. The system implements intelligent result limiting to prevent context window overflow.
2. **Unstructured Retrieval:** Simultaneously, the system performs a vector similarity search using the same embedding model to identify semantically similar clinical text chunks from the vector index.
3. **Context Consolidation:** The results are merged via *context concatenation*. The structured graph outputs (converted to natural language text) and the unstructured vector chunks are combined into a single, unified prompt context window.
4. **Generation:** This unified context is passed to the generation model (GPT-4o-mini) to synthesise the final answer. This model is distinct from the lighter model used for query translation, ensuring the final response prioritizes reasoning capabilities and safety.

#### 3.4.1 Note on source attribution

While the context consolidation approach ensures the model has access to all information, it presents a challenge for precise source attribution. By flattening distinct graph nodes and text chunks into a single context block, the model occasionally struggles to map a specific sentence in the output back to its precise origin node ID, a limitation discussed further in Section 5.

**Figure 4:**
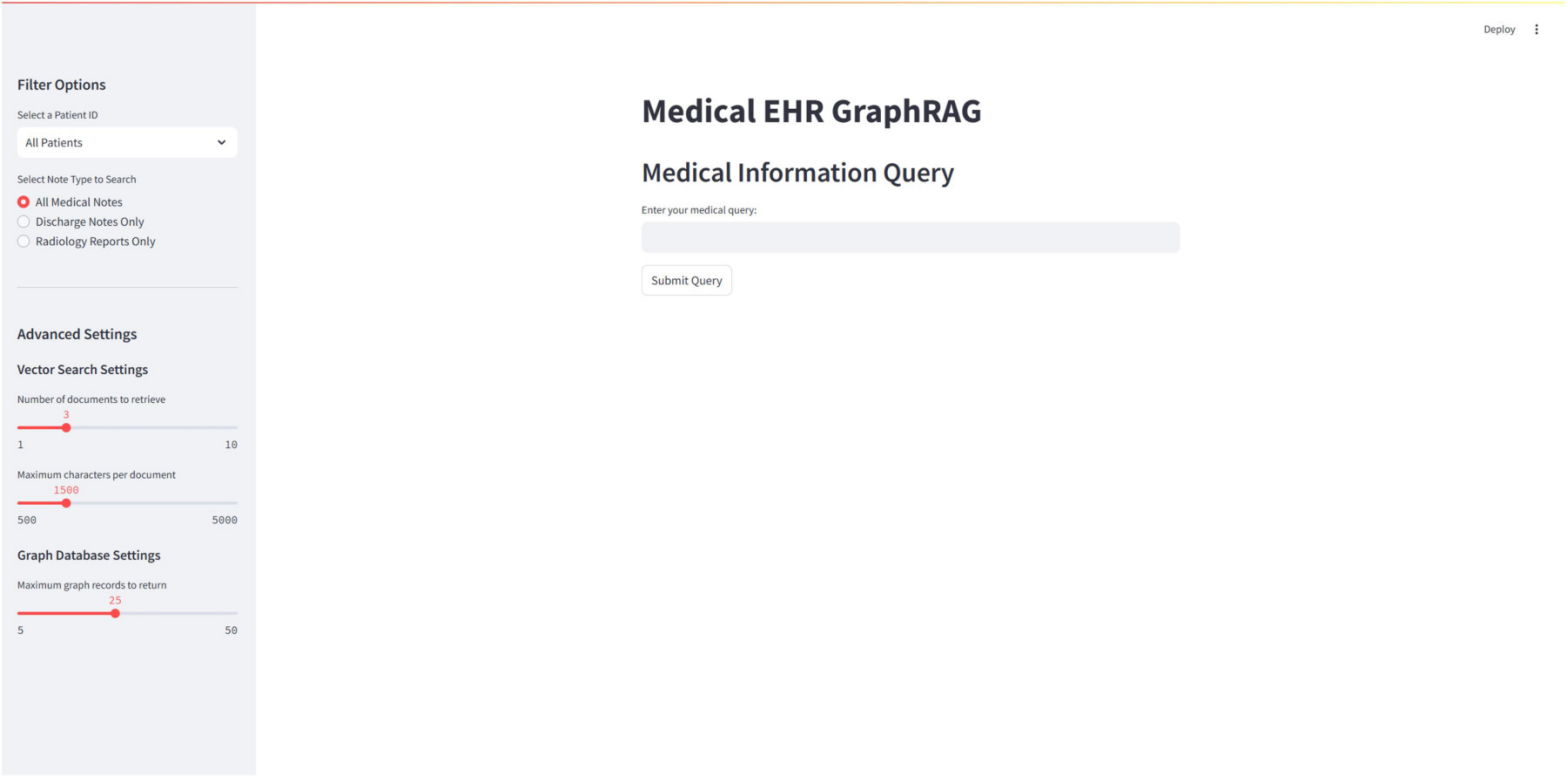
Overview of the MediGRAF system interface showing the main query input area and configurable retrieval parameters. The left sidebar provides filtering options for patient selection, note type filtering, and advanced settings for both vector search (document retrieval limits) and graph database queries (maximum records to return).

The designed architecture was implemented as a fully functional web-based application using Streamlit, providing an intuitive interface for clinical users. Figure 4 shows the implemented system through which all evaluation queries were processed.

### 3.5 Query complexity classification

To evaluate system performance across different challenge levels, queries were classified into three complexity categories. This classification was performed through a manual review process by the research team with domain expert input, defining complexity based on the graph traversal depth and data synthesis requirements.

Simple queries involve direct fact retrieval requiring single node lookups or straightforward relationships, such as patient demographics, admission counts, or document tallies. These queries test the system’s ability to accurately retrieve and present basic clinical facts. Examples include queries such as: “*What is the date of birth for patient 10461137?*” or “*Count the number of admission records.*”

Medium complexity queries require traversal of multiple relationships or temporal reasoning, including medication histories, diagnostic patterns across admissions, or laboratory result trends. These queries assess the system’s capability to integrate information across multiple graph nodes while maintaining temporal coherence. An example of this category is: “*List all medications prescribed to the patient during their admission for pneumonia in 2023,*” which requires linking Admission → Diagnosis → Medication.

Complex queries demand synthesis, summarisation, or inference from multiple data sources, such as comprehensive patient summaries, differential diagnosis reasoning, or treatment response analysis. These queries evaluate the system’s ability to combine structured and unstructured data, perform clinical reasoning, and generate coherent narratives from disparate information sources. A representative example is: “*Summarise the patient’s response to antibiotic treatment across all admissions and identify any recurring complications reported in the discharge notes.*”

### 3.6 Evaluation framework and metrics

The evaluation methodology combined standard information retrieval metrics with clinical expert assessment. The evaluation dataset comprised 141 QA pairs derived from the MIMIC-IV data (30). These pairs were manually curated by three independent medical clinicians and were stratified according to the complexity definitions outlined in Section 3.5. The curators generated questions ranging from simple fact retrieval (Simple) to complex longitudinal reasoning (Complex) questions based on their clinical experience.

Performance was evaluated using distinct methodologies for deterministic (Text2Cypher) and generative (Hybrid) outputs.

#### 3.6.1 Deterministic evaluation (simple/medium queries)

For structured queries where a ground-truth set of records exists (e.g., “Count admissions”), we utilised standard metrics computed as follows (Equation 2):

$$\text{Precision}=\frac{\text{TP}}{\text{TP}+\text{FP}},\;\text{Recall}=\frac{\text{TP}}{\text{TP}+\text{FN}},\;\text{F1-score}=\frac{2\times \text{Precision}\times \text{Recall}}{\text{Precision}+\text{Recall}}$$ (2)

where TP denotes true positives, FP false positives, and FN false negatives.

Additionally, Accuracy is formally defined as the ratio of queries that successfully returned the correct ground-truth answer or result set ($N_{\mathrm{correct}}$) to the total number of queries ($N_{\mathrm{total}}$) (Equation 3):

$$\text{Accuracy}=\frac{N_{\mathrm{correct}}}{N_{\mathrm{total}}}$$ (3)

The metrics are summarised as follows:

- **Accuracy:** The percentage of queries where the generated Cypher returned the exact correct result set.
- **Recall:** The proportion of relevant records retrieved from the database.
- **F1-Score:** The harmonic mean of precision and recall.

For the Hybrid system in Table 3, “Accuracy” represents “Retrieval Success Rate”: the proportion of queries where the relevant information was successfully retrieved into the context window, regardless of the final generation verbosity. This distinction explains why F1-score is marked as N/A for Hybrid approaches, as the generative nature of the output precludes discrete False Positive counting in the same manner as database row retrieval.

**Table 3:** Performance metrics across query complexity levels (N=131).

Complexity	System	N	Accuracy (%)	Recall	F1
Simple	Cypher	100	80	0.8	0.8
Simple	Hybrid	100	100	1.0	N/A
Medium	Cypher	31	51.6	0.688	0.742
Medium	Hybrid	31	100	1.0	N/A

Precision and F1-score are marked N/A for Hybrid approaches because these queries generate generative natural language responses rather than discrete retrieval sets, rendering exact-match metrics inapplicable.

#### 3.6.2 Generative evaluation (hybrid/complex queries)

For complex natural language inquiries where exact-match metrics are inapplicable, we employed a human-expert evaluation protocol. Two independent clinical reviewers (hospital physicians) assessed responses against a gold-standard summary created by a senior clinician. They utilised structured 5-point Likert scales (38) based on the following definitions:

- **Hybrid Accuracy (Likert 1–5):** Defined as the factual correctness of the generated narrative. A score of 5 indicates all medical facts (dates, dosages, diagnoses) are correct compared to the source notes.
- **Completeness (Likert 1–5):** Assesses whether the answer provides all key pieces of information present in the ground truth.
- **Relevance & Conciseness (Likert 1–5):** Assesses if the answer directly addresses the question without redundant or off-topic information.
- **Overall Quality (Likert 1-5):** A holistic judgment of the answer’s clinical utility.
- **Safety (Binary 0/1):** A binary metric where “1” (Unsafe) indicates the presence of a *critical hallucination*: inventing a medical condition, hallucinating a medication not in the record, or contradicting a known contraindication.

Detailed evaluator instructions, scoring rubrics, and the full annotation manual are provided in Supplementary Appendix B. Worked examples illustrating the application of the Safety metric are provided in Supplementary Appendix C.

## 4 Results

### 4.1 Graph database implementation and statistics

The implementation of the MediGRAF system successfully processed data from 10 MIMIC-IV patients, constructing a comprehensive knowledge graph that captured the complexity of their clinical histories. The resulting graph database contained 5,973 nodes distributed across the eight defined node types, with Patient nodes (n=10) serving as central hubs, Admission nodes (n=28) representing distinct hospital encounters, Diagnosis nodes (n=420) capturing the full spectrum of clinical conditions, Procedure nodes (n=29) documenting clinical interventions, Medication nodes (n=1,051) representing pharmaceutical treatments, Lab Event nodes (n=4,346) containing laboratory test results, Discharge Note nodes (n=25) with full clinical narratives, and Radiology Report nodes (n=64) providing imaging insights.

The graph structure comprised 5,963 relationships that encoded the complex web of clinical associations. The relationship distribution revealed the richness of clinical data, with HAS_DIAGNOSIS relationships (n=420), reflecting the diagnostic complexity of hospitalised patients. HAS_MEDICATION relationships (n=1,051) captured the extensive pharmaceutical management, while HAS_PROCEDURE relationships (n=29) documented clinical interventions. The INCLUDES_LAB relationships (n=4,346) connected laboratory results to their clinical context, and HAS_ADMISSION relationships (n=28) maintained the patient-encounter hierarchy. The INCLUDES_RADIOLOGY_REPORT (n=64) connected the patient and their admissions to their unstructured radiology reports and INCLUDES_DISCHARGE_NOTE (n=25) connects them to their discharge summaries.

Vector embedding processing successfully transformed all 89 free-text documents (25 discharge summaries and 64 radiology reports) into searchable vector representations. The embedding process maintained document integrity while enabling semantic search capabilities.

### 4.2 Query performance

Evaluation across three complexity levels demonstrated the system’s robust performance and the complementary nature of graph and vector retrieval mechanisms. High complexity queries were not evaluated using F1, precision, and recall metrics as these queries required multi-source synthesis and inferential reasoning, producing free-text responses without discrete ground-truth answers for comparison. Therefore, high complexity queries were assessed through domain expert evaluation using Likert scales, reported in Section 4.3.

For simple queries (n=100), the Cypher-only system achieved 80% accuracy with matching precision and recall (0.8), yielding an F1 score of 0.8 as shown in Table 3. These queries, which included patient demographics and basic counts, were predominantly answered through Cypher-based graph retrieval. When employing the hybrid approach, the system achieved perfect accuracy (100%) and recall (1.0) which means all relevant facts were retrieved and in the final output; however, precision and F1 metrics could not be calculated as the hybrid system augments responses with contextual information from unstructured sources, producing enriched outputs that extend beyond the discrete ground-truth format required for precision measurement.

Medium complexity queries (n=31) showed more pronounced differences between approaches. The Cypher-only system achieved 51.6% accuracy with precision of 0.806 and recall of 0.688, resulting in an F1 score of 0.742. These queries required integration of multiple data sources, making the hybrid retrieval approach essential. The hybrid system achieved perfect accuracy (100%) and recall (1.0) for medium complexity queries which means all relevant facts were retrieved and in the final output, though precision metrics were similarly not applicable due to the generation of comprehensive, context-enriched responses rather than discrete answers. The performance gap between Cypher-only and hybrid approaches demonstrates the value of incorporating unstructured data for queries requiring multi-source integration.

### 4.3 Clinical expert evaluation

Given that the baseline Cypher-only system was unable to generate valid natural language responses for high-complexity inference queries (frequently returning empty result sets or raw data rows unsuitable for direct clinical comparison), the expert evaluation focused exclusively on the Hybrid system’s outputs. Domain experts assessed 10 complex query responses using Likert scales (1-5, where 5 represents excellent). Table 4 presents detailed statistics for each evaluation dimension. The system achieved high scores for factual accuracy and completeness, with Evaluator 1 rating Accuracy at 4.40±1.02 and Completeness at 4.40±0.92, while Evaluator 2 provided even higher ratings of 4.90±0.30 for both dimensions. Overall Quality scores were consistently strong (4.30±1.00 and 4.20±0.75 for Evaluators 1 and 2, respectively).

**Table 4:** Descriptive statistics for model output evaluation evaluated by clinicians (N=10 items).

Evaluation dimension	Evaluator 1				Evaluator 2			
	Mean	SD	Median	Range	Mean	SD	Median	Range
Accuracy	4.40	1.02	5.0	2–5	4.90	0.30	5.0	4–5
Completeness	4.40	0.92	5.0	3–5	4.90	0.30	5.0	4–5
Relevance & Consciseness	4.20	0.87	5.0	3–5	3.30	0.90	3.0	2–5
Overall quality	4.30	1.00	5.0	2–5	4.20	0.75	4.0	3–5
Safety concerns${}^{a}$	0/10 (0%)				0/10 (0%)			

${}^{a}$Number of items flagged as potentially unsafe out of 10 total items.

All dimensions except safety were measured on a 5-point Likert scale (1 = Poor, 2 = Below average, 3 = Average, 4 = Good, 5 = Excellent).

SD, standard deviation

**Figure 5:**
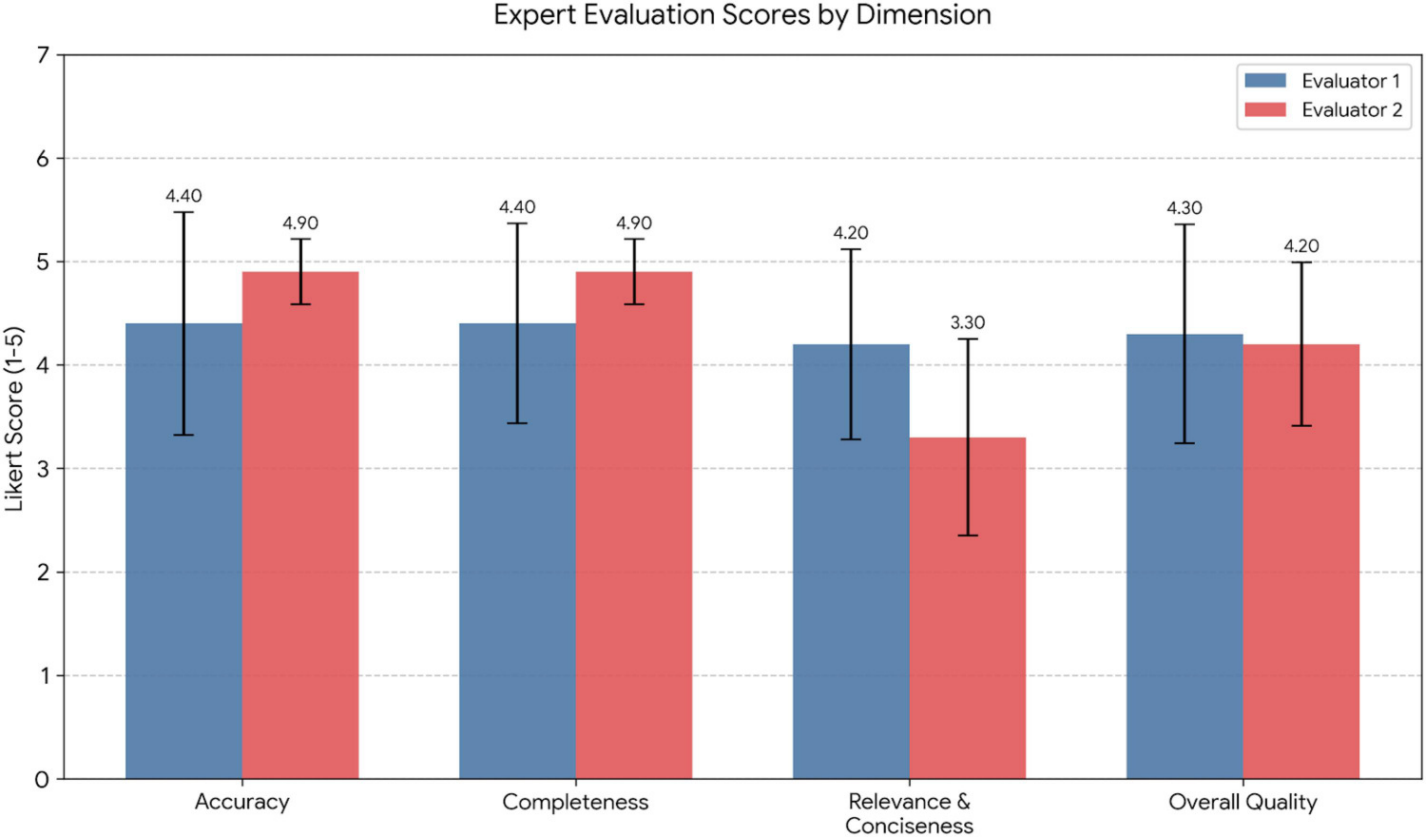
Comparison of mean expert evaluation scores across four dimensions. Error bars represent standard deviation. Note the significant divergence in “Relevance & Conciseness” scores.

Notably, no responses were flagged as potentially unsafe by either evaluator (0/10 cases), addressing a primary concern with LLM applications in healthcare. An interesting divergence emerged in the Relevance & Conciseness dimension, as illustrated in Figure 5. Evaluator 1 assigned a mean score of 4.20(±0.87) while Evaluator 2 was notably more critical at 3.30(±0.90), suggesting that response verbosity remains an area for optimization. This finding was consistent with qualitative feedback from both evaluators.

Qualitative feedback revealed both strengths and areas for improvement. Evaluator 1 praised responses that included “additional details for discharge, such as discharge follow-up plans rather than just answering the primary diagnosis.” However, both evaluators consistently identified verbosity as a recurring issue, with Evaluator 2 noting instances where “the model output is overly verbose for a summary” and Evaluator 1 observing responses that were “very wordy, did not summarise, just pulled straight from clinical notes.”

### 4.4 Error analysis and failure modes

Despite strong overall performance, systematic error analysis revealed specific failure patterns that inform future development priorities. Context window limitations emerged as the primary constraint for hospital-wide queries. Attempts to retrieve multiple queries across all patients resulted in context overflow, as the input tokens exceeded the language model’s processing capacity (128 k tokens for GPT-4o-mini). This limitation necessitated the implementation of intelligent result filtering and pagination strategies for broad-scope queries.

Source attribution occasionally proved challenging when information appeared in multiple contexts. The system sometimes struggled to clearly distinguish between information derived from graph traversal vs. vector search, particularly when both sources contained overlapping information. This ambiguity, while not affecting accuracy, reduced transparency in response generation.

## 5 Discussion

### 5.1 Technical innovation and clinical significance

This research presents a significant advancement in clinical information retrieval through the novel integration of graph database technology with retrieval-augmented generation. The MediGRAF system achieves 100% recall for factual queries while maintaining high performance (mean quality score 4.25/5) for complex inference tasks, demonstrating that hybrid approaches combining Text2Cypher capabilities with vector embeddings are essential for comprehensive EHR querying. Neither technology alone proved sufficient; the graph database excelled at structured relationship traversal while vector search captured semantic similarity in unstructured text.

The system’s architecture addresses critical barriers to clinical adoption through two key innovations. First, the Text2Cypher implementation using GPT-4o-mini eliminates the need for users to learn complex query languages, making graph databases accessible to clinicians. Second, the graph structure provides explicit relationship representation that enables temporal queries and multi-hop reasoning impossible with traditional databases, for instance, queries linking medication changes to laboratory result trends that require understanding both temporal and causal relationships.

**Figure 6:**
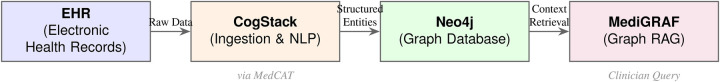
Proposed deployment pipeline illustrating the integration of MediGRAF with the NHS CogStack ecosystem. CogStack handles real-time ingestion and NLP extraction (via MedCAT) to dynamically populate the Neo4j graph, enabling MediGRAF to query live clinical data.

Crucially, the system architecture supports integration with existing NHS data infrastructure, specifically the CogStack platform. While the current evaluation relied on static MIMIC-IV data, the modular design allows MediGRAF to sit downstream of CogStack’s backend data ingestion pipelines demonstrated in Figure 6. This integration provides a pathway to clinical deployment where patient data is ingested, harmonised, and de-identified by CogStack and can dynamically populate the Neo4j graph in near real-time. This bridge between established back end storage and frontend generative AI addresses the challenge of deploying LLMs in live hospital settings.

Furthermore, the choice of a graph-based RAG architecture over a simpler “Prompt-Only” approach (feeding the entire patient record into the LLM) is justified by the specific constraints of longitudinal healthcare data. First, the token volume of a comprehensive patient history spanning years of admissions, hundreds of lab results, and dozens of discharge summaries frequently exceeds the context window limits of modest LLMs. For example, our cohort included patients with up to 9 distinct admissions and 30+ notes (Table 1); processing this raw volume for every query would be computationally prohibitive and induce significant latency. Second, LLMs are known to struggle with arithmetic precision and “needle-in-a-haystack” retrieval within long contexts. By offloading structured retrieval (e.g., “Count all admissions”) to the deterministic Cypher engine, MediGRAF ensures mathematical accuracy that a pure probabilistic model cannot guarantee, effectively solving the trade-off between the comprehensiveness of the patient record and the precision required for clinical safety.

Regarding clinical deployment, the system architecture offers flexible security configurations to meet NHS Trust requirements. While this study utilized secure Azure endpoints, the platform can alternatively be deployed using local, open-source LLMs hosted entirely within a hospital’s firewall, eliminating external API calls where strictly required by data governance policies.

From a clinical perspective, the system directly addresses the problem of information oversight during time-pressured encounters. With NHS clinicians facing increasing patient complexity and documentation burden, the ability to surface all relevant information through natural language queries in sub-second response times could translate to substantial efficiency gains. Most critically, the absence of observed unsafe responses (0/10) achieved validates this approach for mitigating hallucination risks that have limited LLM adoption in healthcare settings.

### 5.2 Performance analysis

The clinical evaluation revealed important insights about system performance across different query types. Simple and medium complexity queries achieved perfect recall, validating the graph database’s ability to capture structured clinical relationships. For complex inference tasks requiring multi-source synthesis, the Likert scale evaluation demonstrated strong performance with high accuracy (4.40–4.90) and completeness (4.40–4.90) scores showing minimal inter-rater variance.

The notable divergence between evaluators on relevance and conciseness (4.20 vs. 3.30) identifies verbosity as the primary area requiring refinement. This 0.90-point difference, the largest observed discrepancy, suggests that while the system successfully captures all relevant information, response optimisation varies significantly based on individual clinician preferences. Importantly, this verbosity issue does not compromise accuracy or safety amongst the 10 patient cases evaluated, the high scores in these critical dimensions confirm that the system reliably synthesizes information without introducing errors or omissions.

The successful processing of the data of the 10 patients in 5,973 nodes and 5,963 relationships with consistently quick retrieval times demonstrates both the scalability potential and the clinical feasibility. These performance characteristics align with recent findings in Hybrid RAG architectures, confirming that hybrid retrieval strategies are necessary for handling the complexity of clinical data.

Our results specifically reinforce the necessity of hybrid retrieval even for queries classified as ’simple.’ As detailed in Table 3, the Cypher-only approach failed to retrieve correct answers in 20% of simple cases, primarily due to gaps in structured coding where clinical signals existed only in free-text narratives. This aligns with recent findings by Sarmah et al. (11], who observed that hybrid systems combining graph traversal with vector search offer superior recall by compensating for the limitations of either modality in isolation. Consequently, we maintain the hybrid architecture for all query types to ensure the system is robust against the data incompleteness inherent in real-world EHRs.

### 5.3 Limitations and mitigation strategies

Several limitations require acknowledgment while recognising their addressability. The evaluation scope of 10 patients assessed by two evaluators, while appropriate for proof-of-concept validation, limits generalisability claims. The observed inter-rater variability, particularly in subjective dimensions, underscores the need for larger evaluator cohorts and standardized assessment criteria. Future studies should include more clinical experts and patients from multiple institutions to establish robust reliability metrics.

The verbosity issue, while not affecting accuracy, could impact usability in time-pressured environments. This challenge is addressable through prompt engineering refinements including response summarisation layers, adjustable verbosity settings, and context-aware tailoring based on query urgency. The context window limitations for hospital-wide queries can be mitigated through query decomposition and streaming architectures or the use of different models which have larger context windows.

The use of Likert scales for complex query evaluation, while subjective, was necessary given the absence of discrete ground truth for inference-based responses. This methodological choice aligns with established practices in clinical NLP evaluation. The MIMIC-IV dataset’s single-institution origin requires acknowledgment, though its comprehensiveness provides a strong foundation for initial validation.

Finally, regarding interpretability, the current architecture utilizes a “context flattening” strategy where structured graph outputs and unstructured vector chunks are merged into a single prompt window. While this maximizes the information available to the LLM, it complicates precise source attribution. Unlike citation-aware models that generate granular footnotes linking specific claims to specific source nodes, our current approach provides document-level provenance but lacks token-level attribution statistics. Future iterations will address this by implementing citation-aware generation layers to allow for rigorous statistical quantification of source usage (e.g., measuring the ratio of graph-derived vs. vector-derived facts in the final output).

### 5.4 Conclusion and future directions

This research establishes MediGRAF as a viable solution for clinical information retrieval, demonstrating that combining graph databases with large language models creates capabilities beyond either technology alone. The integration of Model Context Protocol (MCP) and agentic workflows represents the natural evolution, enabling more sophisticated multi-step reasoning and dynamic query refinement (39].

The path forward requires systematic expansion from proof-of-concept to clinical deployment. Prospective trials measuring time savings, decision quality, and user satisfaction in actual clinical settings will validate practical impact. While the zero incidence of unsafe responses in this preliminary cohort (n=10) is encouraging, it serves primarily to justify proceeding to larger-scale trials where safety can be evaluated across a broader range of clinical contexts and specialities.

For healthcare systems facing mounting pressure on clinical resources, this technology offers a template for responsible AI deployment that maintains factual grounding while providing natural language accessibility. By demonstrating feasibility, safety, and performance advantages, this work contributes three key advances to health data science: validation of graph-based EHR representation benefits, evidence for hybrid retrieval superiority in healthcare contexts, and a framework for safe LLM deployment through factual grounding. As healthcare continues its digital transformation, such systems will become increasingly critical for managing clinical data complexity while maintaining the human-centered focus essential to quality patient care.

## Data Availability

Publicly available datasets were analyzed in this study. This data can be found here: https://physionet.org/content/mimiciv/3.1/hosp/#files-panel.
